# Cefepime versus Ceftriaxone for perioperative systemic antibiotic prophylaxis in elective orthopedic surgery at Bugando Medical Centre Mwanza, Tanzania: a randomized clinical study

**DOI:** 10.1186/s40360-015-0039-4

**Published:** 2015-12-23

**Authors:** Joel M. Marwa, Isidor H. Ngayomela, Jeremiah Seni, Stephen E. Mshana

**Affiliations:** Department of Surgery, Catholic University of Health and Allied Sciences, Box 1464, Mwanza, Tanzania; Department of Trauma and Orthopedics, Bugando Medical Centre, Box 1370, Mwanza, Tanzania; Department of Microbiology/Immunology, Catholic University of Health and Allied Sciences (CUHAS), P.O. BOX 1464, Mwanza, Tanzania

**Keywords:** Surgical Site Infection, Ceftriaxone, Cefepime, Mwanza, Tanzania

## Abstract

**Background:**

Antimicrobial prophylaxis reduces the incidence of postoperative wound infections especially among patients undergoing orthopedics surgery. However, there is dearth of information on the clinical effectiveness, spectrum limitations and practical contextual information on third and fourth generation cephalosporins. The aim of this study was to evaluate the efficacy and safety of cefepime and ceftriaxone as peri-operative systemic antimicrobial prophylaxis in elective orthopedic surgery in our center.

**Methods:**

This study was a prospective, randomized, open label comparative clinical study of patients undergoing elective orthopedic procedures at the Bugando Medical Centre (BMC) between June 2014 and February 2015. Two hundred thirty participants were enrolled in the study and randomly assigned into Ceftriaxone regimen (group A) or Cefepime regimen (group B). Participants in ceftriaxone or cefepime group received 50 mg/kg up to 2 g single dose perioperative intravenous infusion at least 30 min before incision. Both groups were followed for 30 days using a Center for Disease Control superficial surgical site infection criterion for the outcome. A two-tailed margin of equivalence was set at 5 % analyzed on the intent to treat.

**Results:**

All 230 participants were subjected to final analysis with no patient being lost to follow-up. Superficial surgical site infection occurred in 5 out of 117 (4.3 %, 0.6 to 7.9 at 95 % CI) patients receiving cefepime compared to 3 out of 113 (2.7 %, 0.3 to 5.6 at 95 % CI) among patients receiving ceftriaxone regimen. The absolute difference of 1.6 % (95 % Confidence Interval: −6.3 to 3.1), equivocally lies outside the 5 % statistically significant margin of presumed clinical equivalence.

**Conclusion:**

The difference between cefepime and ceftriaxone in preventing SSIs following elective clean orthopedic surgery was not statistically significant.

**Trial Registration:**

Pan African Clinical Trial Registry: PACTR201406000803420

## Background

Surgical site infections (SSIs) continue to be a major source of morbidity and mortality in developing and resource limited countries despite advances in aseptic techniques [1–4]. Orthopedic patients especially for those who require corrective surgical procedures have a greater risk of surgical site infections [5]. SSIs are important complications of orthopedic procedures often associated with prolonged length of hospital stay, high incidence of readmission, huge treatment costs and low quality of life [6].

The principles of prophylaxis against post-surgical infection have been established and the administration of antibiotics within 60 min prior to surgery is now widely accepted [7]. The choice of perioperative prophylactic antibiotics follows the principles that the selected regimen should target microbial agents commonly involved in surgical site contamination and definite infection. In orthopedics surgery seeding of the operative site from a distant site of infection can also occur especially in patients with prosthesis or other implants [8].

Evidence has shown that a single dose cephalosporin prophylaxis leads to a significant reduction in the proportion of developing surgical site infections, and is adequate in orthopedic surgical procedures [9–12]. A latest Cochrane systematic review reported that perioperative prophylaxis during the operative management of closed fractures reduce infection rates from around 5 % to less than 1 % [13].

Recently, after the introduction of cefepime in Tanzania, clinicians started prescribing it in the place of previously favored regimes such as ceftriaxone, penicillins and aminoglycosides [14]. However, at Bugando Medical Centre (BMC), the prophylactic efficacy of cefepime over other cephalosporins or other prophylactic regimens remains unknown, especially in our trauma and orthopedic surgery.

No standard or written guidelines exist in our centre to direct the choice of effective or appropriate antimicrobial regimen for use as perioperative prophylaxis during elective orthopedic surgery. Therefore, this study was performed to compare the efficacy of cefepime and ceftriaxone for systemic perioperative prophylactic use in elective orthopedic surgery at BMC.

## Methods

### Study design

This study was a comparative, open label, prospective randomized clinical trial. It was performed to compare the efficacy, of a single dose prophylactic cefepime and ceftriaxone among patients underwent elective orthopedic surgery at BMC, Mwanza Tanzania.

### Study population

The study population included all patients admitted at the BMC orthopedic wards and planned for elective orthopedic surgical procedure between June 2014 and February 2015 [15]. All orthopedic patients between 1 and 70 years of age planned for elective orthopedic surgery were considered eligible. However, patients with open contaminated fractures, history of any antibiotic use within 7 days preceding surgery, known history of hypersensitivity to beta-lactams, immunodeficiency disorders, HIV infection, pregnancy, diabetes or existing infection of soft tissue, bone or at the site of the fracture were excluded from the study.

### Sample size estimation

The sample size was estimated using the formula published by Altman [16] and as used by Noordzij et al. [17]. The expected SSI rate estimates for ceftriaxone and cefepime were 2.3 % and 1.1 % respectively among patients as published by Del Rio et al. [18]. The conventional multipliers for alpha = 0.05 and multiplier for power = 0.90 were used and we considered a pre-study difference of more than 5 % between the two groups to be statistically significant. The sample size obtained was 208 patients (a minimum of 104 participants per group) and 10 % of this estimate was added to cover for non-response or loss to follow up, thus the required minimum sample size became 230 patients.

### Randomization

Participants were assigned to one of two treatment groups, designated as “A” (ceftriaxone) and “B” (cefepime) using four digits, randomly generated computer numbers. Randomization to the two study arms was at 1:1 ratio. Each patient received 50 mg/kg (maximum 2 g) intravenous antibiotic given within 30 min before surgery. In case surgery lasted beyond 4 h or blood loss, surpassed 1500 mL the dose was repeated.

### Null hypothesis

The treatment difference on the proportion of elective orthopedic surgery SSI in the two arms should be less or equal to ± 5 %.

### Alternative hypothesis

The treatment difference on the proportion of elective orthopedic surgery SSI in the two arms should be more than ± 5 %.

### Explanatory variables

Independent variables were demographic data (e.g. age, sex, and occupation), clinical presentation, ASA classification, type of orthopedic surgical procedure and implants used. In addition, duration of procedure, blood loss, type of anesthesia, wound closures, placement or removal of internal or external implants, and use of drains were also included.

### Primary outcome measure

Surgical site infection was the primary end point carried out between day 3 and 30 after surgery. Two members of the orthopedic team alien to the study provided surveillance and clinical diagnosis of surgical site infection in the ward or SOPD. The CDC criteria of wound infection occurring at the incision site within 30 days after surgery and involving the skin, subcutaneous tissue, or muscle located above the fascial layer formed the basis for SSIs surveillance was used [19].

Surveillance continued for 30 days by making telephone calls and if patients reported any symptoms of wound infection patients were requested to return to the hospital for re-examination and specimen collection.

### Microbiology laboratory studies

Wound discharge or pus were collected from infected surgical incisions using sterile cotton swabs without contaminating with skin commensals and was placed in a sterile bottle with transport media (Oxoid, UK). Collected samples were transported to laboratory within 1 h after being obtained. In the laboratory, specimens were registered and processed following standard laboratory procedures (SOPs) [20].

Isolates were identified using in-house biochemical tests as previously described [21, 22]. All isolates were subjected to antimicrobial drug susceptibility testing using disk diffusion method as stipulated by the Clinical Laboratory Standard Institute (CLSI) guidelines [23].

### Data collection

Data were recorded on three different data sheets coded as Data Sheets A, B, and C. Data Sheet A focused on Contact information, socio-demographic information of the patient, morbid history and preoperative laboratory test results. The explanatory data captured included: age, gender, mechanism of injury, time to treatment, medical history and prior treatments, co-morbidities, smoking, alcohol use, andrecent history of antibiotic use. The attending doctor collected on or near the day of admission this information.

Sheet B contained intra-operative data regarding the treatments option for each injury, including the dates of the treatments, types of procedures, operative time, and blood loss, type of anesthesia, wound closure options, placement or removal of internal or external implants. This information on data sheet B was collectedintra-operatively corroborating with real time medical records by anesthetic and nursing staff.

Data Sheet C contained postoperative surveillance and follow-up information regarding progress, and subsequent treatment outcomes. In addition, the PI and researchassistants did transcribed phone conversations and completed progress charts during scheduled SOPD clinic visits.

### Data management and statistical data analysis

Data were entered and cleaned using SPSS® version 21 (IBM Corporation) and re-assigned into STATA® Version 11 for analysis. The analysis of data involved hypothesis testing and comparison of study outcomes between cefepime and ceftriaxone groups. A statistical difference was established for a significant difference in cumulative incidence of surgical site infection between patients undergoing elective orthopedic surgery receiving peri-operative e ceftriaxone and cefepime regimen. Four parameters- cumulative incidence, incidence rate, 95 % confidence intervals and *P-*values were used to delineate results. Fisher’s Exact Test and *P*-value of analyzed data < 0.05 was considered to be statistically significant.

Cumulative incidence was calculated as the proportion of cases with SSI noted over total number of participants in each regimen under study. Incidence rate was computed by dividing the number of study subjects manifesting wound infection with total person follow-up days. Proportion test was done to determine the 95 % CI of the rates of SSI in two arms.

Data were summarized in form of proportions, and frequency tables, for categorical variables, while measures of central tendency were used to summarize continuous variables.

### Ethical clearance

This trial did not involve new drugs but only determined the efficacy of a single dose regime of ceftriaxone and cefepime, however GCP and Declaration of Helsinki were observed. The study was cleared by CUHAS/BMC Research Ethics Committee with certificate no CRE/010/2014. An informed consent was obtained from participants after explaining the rationale of the study.

## Results

### Number of patients recruited

**Fig. 1:**
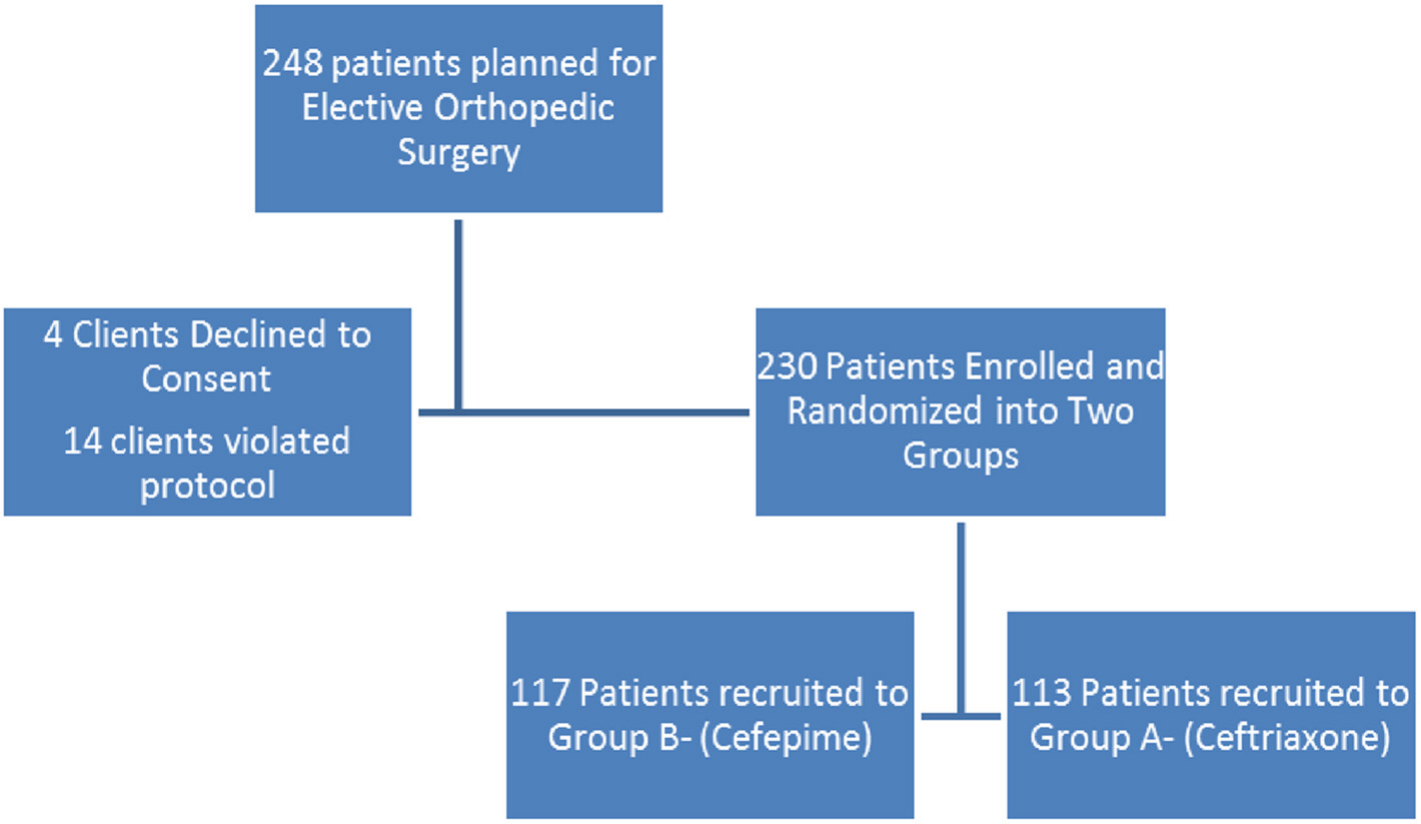
Flow chart showing disaggregated number of participants

A total of 248 patients who were planned to undergo elective orthopedic surgery were assessed for eligibility between June 2014 and February 2015. Fourteen were excluded for failure to meet the inclusion criteria, while four candidates declined to consent for enrollment into the clinical trial. Consequently, 230 patients were recruited and randomized into ceftriaxone (Group A, 113 patients) and cefepime (Group B, 117 patients) as shown in Fig. 1.

### Demographic characteristics of the study population

There was an even distribution of the participants with respect to demographic and other baseline characteristics in group A and group B (*p*-value >0.05) as shown in Table 1.

**Table 1:** Background epidemiological parameters of patients issued with single dose antibiotic prophylaxis during elective orthopedic surgery

Variable	Ceftriaxone (Group A) *N* = 113	Cefepime (Group B) *N* = 117	*p*-value
Age (years)			
Median	29	30	0.3229
IQR	14–40	21–40	
Age group			
<21 years	40 (35.40 %)	29 (24.79 %)	0.292
21–40 years	48 (42.48 %)	60 (51.28 %)	
41–60 years	23 (20.35 %)	24 (20.51 %)	
>60 years	2 (1.77 %)	4 (3.42 %)	
Sex			
Males	77 (68.1 %)	81 (69.2 %)	0.562
Females	36 (31.9 %)	32 (30.8 %)	
Level of education			
Primary	56 (49.6 %)	56 (47.9 %)	0.859
Secondary	44 (38.9 %)	48 (41.0 %)	
Tertiary	9 (8.0 %)	8 (6.8 %)	
Informal	4 (3.5 %)	5 (4.3 %)	
Occupation			
Employed	38 (33.63 %)	39 (33.33 %)	0.095
Unemployed	35 (30.97 %)	50 (42.74 %)	
Dependant	40 (35.40 %)	28 (23.93 %)	
Mode of Health Care Financing			
Out of Pocket	90 (79.65 %)	91 (77.78 %)	0.729
Health Insurance	23 (20.35 %)	26 (22.22 %)	

### Pre-operative clinical presentation

Even distribution of pre-operative clinical characteristics was observed in both arms except for site of lesion and time taken before definitive treatment was given (*p*-value < 0.05) as seen in Table 2.

**Table 2:** Pre- operative parameters of patients subjected to prophylactic single dose regimen during elective orthopedic surgery

Variable	Ceftriaxone (Group A)	Cefepime (Group B)	*p*-value
	*N* = 113	*N* = 117	
Indication for surgery			
Trauma related	91 (80.53 %)	100 (85.47 %)	0.318
Non-Trauma Related	22 (19.47 %)	17 (14.53 %)	
Anatomical location of lesion			
Upper Limb	44 (38.9 %)	30 (26.6 %)	0.031
Lower limb	69 (61.1 %)	87 (74.4 %)	
Time to treatment			
Less than 2 weeks	29 (25.7 %)	48 (41.0 %)	0.014
More than 2 Weeks	84 (74.3 %)	69 (59.0 %)	
History of antibiotic use			
None	84 (74.3 %)	81 (69.2 %)	0.39
More than 7 days	29 (25.7 %)	36 (30.8 %)	
Type of elective procedure			
Invasive	93 (82.3 %)	104 (88.9 %)	0.154
Non Invasive	20 (17.7 %)	13 (11.1 %)	

### Intra-operative clinical presentation

No statistical differences were observed in the distribution of intra-operative clinical characteristics in both groups as seen in Table 3.

**Table 3:** Selected intra-operative considerations of patients subjected to single dose regimen during elective orthopedic surgery

Variable	Ceftriaxone (Group A)	Cefepime (Group B)	*p*-value
	*N* = 113	*N* = 117	
Hemoglobin Level			
Mean	11.6 g/dl	11.3 g/dl	0.577
Range	6–17.2 g/dl	5.8–16 g/dl	
SDEV	2.17 g/dl	2.10 g/dl	
Timing of prophylaxis [within]			
<15 min	65 (56 %)	57 (48.7 %)	0.408
>16– < 30 min	43 (38.0 %)	54 (46.2 %)	
>31– < 45 min	5 (6.0 %)	6 (5.1 %)	
>46 < 60 min	0	0	
Approximate blood loss			
Less than 400 ml	93 (82.3 %)	88 (75.2 %)	0.189
More than 400 ml	20 (17.7 %)	29 (24.20 %)	
Duration of operation [within]			
<1 h	60 (53.1 %)	58 (49.6 %)	0.864
2 h	43 (38.1 %)	45 (38.5 %)	
3 h	9 (8.0 %)	13 (11.1 %)	
4 or More hours	1 (0.8 %)	1 (0.8 %)	
ASA Classification			
ASA 1	105 (92.92 %)	102 (87.18 %)	0.116
ASA 2	7 (6.19 %)	15 (12.82 %)	
ASA 3	1 (0.88)	0.00	

### Clinical outcomes

Increasing tenderness at surgical site after discharge from ward was reported among 3 (2.6 %) patients in cefepime group and 1 (0.9 %) patient in the ceftriaxone arm. Four clients in-group B demonstrated fever > 38.5 °C during the follow up period while 112 (99.1 %) in group A and 112 (95.7 %) in-group B schedule did not report any event during routine enquiry or follow up Table 4.

**Table 4:** Selected post-operative characteristics in patients subjected to perioperative single dose regimen

Variable	Ceftriaxone (Group A)	Cefepime (Group B)	*P*-value
	*N* = 113	*N* = 117	
Increasing tenderness			
Absent	111 (98.23 %)	113 (96.58 %)	0.81
Present	1 (0.9 %)	3 (2.6 %)	
Indeterminate	1 (0.9 %)	1 (0.9 %)	
Morbid fever			
Absent	112 (99.12 %)	112 (95.73 %)	0.122
Present	0 (0.00 %)	4 (3.43 %)	
Indeterminate	1 (0.9 %)	1 (0.9 %)	
	0	0	
Wound dehiscence			
Present	3 (2.65 %)	5 (4.27 %)	0.380
Absent	110 (97.35 %)	112 (95.73 %)	
Occupied Bed Days			
1–5 days	68 (60.2 %)	58 (49.6 %)	0.211
6–10 days	43 (38.0 %)	45 (38.5 %)	
11–15 days	2 (1.80 %)	13 (11.1 %)	
Primary outcome (SSI)			
Absent	110 (97.35 %)	112 (95.73 %)	0.380
Present	3 (2.65 %)	5 (4.27 %)	

### Incidence of SSI in elective surgery

**Fig. 2:**
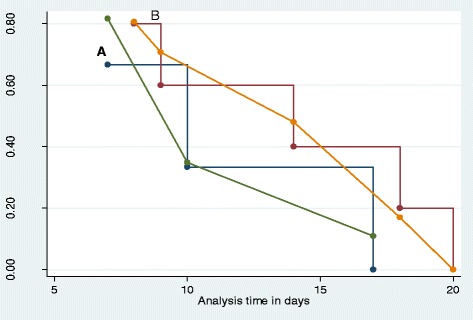
Kaplan-Cox-Meier probability estimates of developing (surviving) superficial surgical site infection in elective orthopedic surgery **a**: Ceftriaxone, **b** Cefepime

Among 230 clients 8 (3.47 %) developed superficial surgical site infections. The disaggregated incidence in the ceftriaxone group was 2.7 % [95 % CI 0.3–5.6) compared to 4.3 % (95 % CI 0.6–7.9) for patients enrolled into the cefepime group (*P =* 0.380) as shown in Table 4. The probability of developing superficial SSI decreased over time manifested as wound dehiscence and positive culture as shown in Kaplan Cox and Meier survival graph (Fig. 2).

### Efficacy of cefepime over ceftriaxone regimen

The incidence rate was calculated as person days for both arms of clinical study. In this study the incidence rate of superficial surgical site infection were 0.9 and 1.45 per 1000 person days in group A (ceftriaxone) and group B (cefepime) regimes respectively (*p* = 0.380) Table 5.

**Table 5:** Rate of superficial surgical site infection and drug efficacy in elective surgery

Study group	Number evaluated	Number infected	‘000 person days	IR	IRR (95 % CI)	*P*- value
Group A	113	3	3324	0.9	0.62 (0.26–1.37)	0.318
Group B	117	5	3429	1.5		

*IR* Incidence Rate (i.e. number per 1000 person days), *IRR* Incidence Rate Ratio

### Bacterial isolates

As empirical proof of infection, bacterial isolates were necessary and useful in the choice of antimicrobial among patients presenting with clinical features of SSIs. The bacteriological isolates in the ceftriaxone group were *Staphylococcus aureus, Pseudomonas aeruginosa* and *Coagulase negative Staphylococcus*. In-group B (cefepime) regimen, bacteriological isolates were *Staphylococcus aureus*, Coagulase *negative Staphylococcus, Proteus* spp., *Klebsiella pneumoniae* and 1 unidentified gram negative bacteria.

All bacterial isolates in ceftriaxone and cefepime groups were sensitive to ciprofloxacin, ceftazidime and gentamicin. The *Proteus* spp*.*isolated was resistant to ceftazidime, amoxicillin clavulanic acid and Trimethoprim/sulphamethaxazole (TMP/SMX).

## Discussion

### Demographic and clinical characteristics of patients

The contribution of antibiotic single agent perioperative prophylaxis to reduce SSIs for most surgical procedures is established. However, optimal use of prophylactic antimicrobial agents does not obviate other factors such as meticulous attention to basic infection-control strategies. The present study did not define age, gender, or mechanism of injury as significant risk factors associated with SSIs. These findings are in agreement with Maksimovic et al. [2] and Graf et. al [24] who found no significant association in age or gender between case patients and their matched interventions. However, in a recent Egyptian study, it was reported age above 68 years to be a risk factor for SSIs among 93 orthopedic patients [25]

Recently, the contribution of prolonged pre-operative duration hospital stay of 13.6 ± 1.6 days and duration of operation beyond 75^th^ percentile for procedure have been strongly linked to increase SSI in orthopedic surgery (Khaleid et al. [25]. In the present study, there was a statistically significant difference in participants distribution in two groups regarding time spent in hospital before treatment and the anatomical site of the lesion. Despite majority of patients in ceftriaxone group stayed significantly longer in the hospital before treatment less SSI was observed in this group. Overwhelmed surgical systems and resource poor setting may explain prolonged hospital stay whereas lower limb lesions pre-dominate since it is the most common injury incurred by motorcycle accidents victims in our setting [26].

### Incidence of superficial surgical site infection

This study demonstrated that the cumulative incidence of superficial SSI after intervention among the participants was comparable to rates detected in some developing countries [4, 27–29]. While in this study ceftriaxone arm showed a lower cumulative incidence compared to cefepime arm, the difference was not statistically significant. These findings are in tandem with landmark studies and meta-analysis reviews that pegged effects of a single agent perioperative prophylaxis in reduction of SSI incidence rate to a range between 0 to 8 % among patients who underwent elective orthopedic surgery. Comparatively, and in consideration of cefepime and ceftriaxone head-to-head non-orthopedic comparative multicenter trial in Parma Italy the study reported the SSI incidence of 2.3 % for ceftriaxone group and 1.1 % in the cefepime group. However, the difference of SSIs among 209 participants was not statistically different [18]. Using similar methodology in the present study, the incidence nearly doubled, which speculatively may be attributed to wound class and target organism selectivity in orthopedics compared to biliary surgery. However, authors in the Italian study attributed the apparently low SSI incidence to a zwitterionic oxymino β-lactam amino-thiazole side chain- a chemical peculiarity- of cefepime rather than other study factors.

The present study also showed a low cumulative SSI incidence in contrast to the outcomes of recent descriptive studies, which reported high SSIs cumulative incidence within disaggregated wound class. Recently in Egypt, Khaleid et al. [25] reported an cumulative orthopedic SSI incidence rate of 25.8 % (4.1 % in clean wounds). More so, Maksimovic et al. [2] and Graf et al. [24] reported an overall orthopedic SSI incidence of 22.7 %(13.2 % in clean wounds) and 22.5 % respectively in their studies. These studies among many other reasons attributable to higher incidences of orthopedic SSI reported failed tostate or show whether the researchers used perioperative prophylaxis among the participants.

### Comparison of single dose efficacy between cefepime and ceftriaxone

This study showed a lower incidence rate of SSIs per 1000 days in ceftriaxone group and nearly twice the rate in cefepime group, although the difference was not statistically significant. The difference in the incidence rate per 1000 days in the cefepime group could not be attributed or explained on theoretical grounds or its molecular profile -as a fourth generation cephalosporins the same as speculated by Yeap et al. [30]. The spectrum differences may not explain the difference since both have a near preponderance to gram-negative organism.

The Incidence Rate Ratio of less than 1.0 was observed in this study and underscored an overall picture of substantial effect of cefepime and ceftriaxone in reducing the incidence of clean orthopedic surgical site infection, despite of spectrum and selectivity limitations. Other studies conducted involved comparison between second or third generation cephalosporins and documented similar trends on the reduction of SSI incidence rate in orthopedic surgery [8, 31, 32]. However, there might be fewer studies published comparing cefepime with other generations of cephalosporins, despite its documented use as perioperative prophylaxis during orthopedic surgery [3, 30].

Ceftriaxone and cefepime are both known to have excellent bioavailability and in this study, a single dose of 50 mg/kg in pediatric population and up to 2 g intravenous infusion among adults was given optimally. The cost implication between cefepime and ceftriaxone was vast and at the time of this study, the cost of 2 g ceftriaxone was USD 1.20 compared to USD 12.80 (ten times less) for 2 g of Cefepime. Therefore, the acquisition cost of ceftriaxone was quite substantially lower than cefepime as a single dose prophylaxis.

The findings also compares well to a Cost Benefit Analysis study by Mazza [33] involving 477 consecutive patients who received ceftriaxone before undergoing orthopedic surgery which reported that a single dose ceftriaxone given prophylactically was a cost-effective measure compared to placebo based on infection incidence and length of hospital stay.

### The etiological agents of superficial SSI in ceftriaxone and cefepime groups

In the present study, it was observed that the predominant organisms causing SSIs after clean procedures in ceftriaxone group were *Staphylococcus aureus*, Coagulase-negative staphylococci*,* and*Pseudomonas aeruginosa*. While in cefepime regimen, bacteriological isolates were *Staphylococcus aureus*, *Coagulase negative Staphylococcus, Proteus* spp., *and Klebsiella pneumoniae.* These findings were congruent with studies conducted at BMC by Mawalla et al. [1] and Khaleid et al. [25]. A similar observation was made by USNational Healthcare Safety Network, January 2006-October 2007 while reporting on distribution of pathogens related to orthopedic surgery [34].

The isolation of *Staphylococcus aureus* among both study groups is in agreement with observations made by the New York State 2009 report where *Staphylococcus aureus* accounted for 59.8 % of total isolates in orthopedic surgical site infections [35]. A possible explanation for *Staphylococcus aureus* being a dominant cause ofwound infection in orthopedic surgeryis generally correlated to admission from a healthcare facility and nasal carriage of *Staphylococcus aureus* [36].

During this study infection due to other *Staphylococcus* spp., isolates had a late presentation post operatively concurrently with *Pseudomonas aeruginosa*; a trend also noted by Moss et al. [37]. The drug sensitivity and resistance pattern of bacterial isolates did not differ among groups and were broadly resistant to β-lactams, and were all sensitive to gentamicin and ciprofloxacin.

## Conclusion

Though relatively more SSIs were observed when single dose of cefepime was used for prophylaxis in elective orthopedic surgery compared to ceftriaxone this was not statistically significant. *Staphylococcus aureus* and gram-negative bacteria depicting resistance to β-lactam based antibiotics are the dominant etiological agents causing superficial surgical infection in elective orthopedic surgery at BMC.

## Abbreviations

BMC: Bugando Medical Centre
CDC: Centre for Disease Control
SSI: Surgical Site Infection
SOPD: Surgical Outpatient Patient Department
PI: Principal Investigator
CUHAS: Catholic University of Health & Allied Science
GCP: good clinical practice
